# Communicating Effectively With Limited English Proficiency Patients: Incorporating Training on Working With Medical Interpreters in the Undergraduate Medical Curriculum

**DOI:** 10.7759/cureus.75207

**Published:** 2024-12-06

**Authors:** Algevis Wrench, Lauren Fine, Daniel Griffin

**Affiliations:** 1 Medical Education, Nova Southeastern University Dr. Kiran C. Patel College of Allopathic Medicine, Fort Lauderdale, USA

**Keywords:** cultural competency, health disparities, limited english proficiency, medical spanish, professional medical interpreter, undergraduate medical education

## Abstract

Introduction: It is imperative for the healthcare providers in the United States to be able to care for the growing number of patients with limited English proficiency (LEP) utilizing professional medical interpreters (MIs). Since little time in undergraduate medical education (UME) is devoted to this competency, an educational workshop on effective communication with MIs and Spanish-speaking LEP patients was developed.

Methods: A two-hour workshop was implemented for first-year medical students, featuring four educational strategies: (1) facilitator-led instructional simulation, (2) interactive didactic, (3) small-group clinical case discussion, and (4) large-group MI simulation. Participant volunteers completed anonymous, pre-/post-workshop evaluations and self-assessments on their knowledge of factors that impact medical interpretation skills.

Results: Of 51 participants, 43 (84%) completed the pre- and post-workshop evaluations. Approximately 83% reported “little to no” or “limited” Spanish language fluency. Participants reported a statistically significant (p<0.05) increase in confidence to explain the importance of using MIs to improve LEP patient care, and to demonstrate both respect and cultural humility when using MIs. The self-assessment showed an increase in average scores for all questions tested. Ninety-eight percent of the participants strongly agreed or agreed that the workshop met its objectives. Thematic analysis of qualitative feedback centered around three major themes: MI need, MI practice, and medical Spanish knowledge.

Conclusion: This curricular intervention successfully increased self-reported measures of skills and best practices for communicating effectively with Spanish-speaking LEP patients utilizing MIs. The implementation of MI training programs in UME can be a successful strategy to prepare trainees to provide care for the growing LEP population.

## Introduction

Effective communication between healthcare providers and patients is essential to the provision of safe and high-quality patient-centered healthcare [1]. Patients with language barriers experience health disparities including higher risk of non-adherence to treatment recommendations and receipt of inadequate information on disease prevention, disease state, and medical consent [2-3]. They also have a greater dissatisfaction with care and are at a higher risk of medical errors resulting in physical harm [4-5]. In 2020, people with limited English proficiency (LEP), defined by the United States (US) census as those who speak English less than “very well,” represented 8.2% (25.3 million) of the US population aged five years old and older [6]. About 21.5% (66.1 million) of the US population speaks a language other than English, with Spanish being the most common language spoken at home [6]. In Florida, 21.8% (4.4 million) of the population speaks Spanish at home [6]. The above-mentioned factors highlight the importance of improving communication in healthcare settings to bring better health outcomes.

A professional medical interpreter (MI) can minimize the health disparities experienced by LEP patients. Studies have consistently shown that the use of MIs is associated with enhanced communication between LEP patients and providers as well as improved health processes and outcomes. Some benefits include improved comprehension, fewer communication errors, lower risk of adverse effects, and increased patient satisfaction [7]. Despite federal mandates for provision of healthcare services in a patient’s language by means of qualified professionals, as part of Title VI of the 1964 Civil Rights Act, the use of family members or untrained staff as ad hoc interpreters remains widespread and presents serious patient safety risks [8-9].

Despite the growing awareness of adverse health outcomes for the LEP patients and increasing numbers of non-English-speaking patients seen by providers, there is generally little curricular time in medical training devoted to the effective use of MIs. In 2017, only 29 of 147 Liaison Committee on Medical Education (LCME)-accredited medical schools offered a formal curriculum that taught medical students how to work with MIs and/or patients with LEP [10]. Many of these curricular activities that concentrate on the improved use of MIs have focused on web-based or discussion-based practices [11-12]. While these approaches are useful, they lack the educational benefits provided by the use of role play, simulation, reflection, and feedback. Engaging in a skill through role play has been shown to increase confidence with the skills practiced in various fields including the use of professional medical interpretation [13-15].

As the LEP population continues to grow, it is imperative that medical students become aware of the importance of medical interpretive services and become more skilled in the use of MIs to improve patient care for the LEP population. To address the need for incorporating the effective use of professional MIs in undergraduate medical education (UME), we developed an educational workshop designed to provide medical students with skills and best practices on how to communicate effectively with MIs and Spanish-speaking LEP patients.

This article was previously posted to the Research Square preprint server on October 28, 2022.

## Materials and methods

Ethical approval and curriculum implementation

This study was reviewed and approved with exempt status in March 2022 by the Nova Southeastern University Institutional Review Board (IRB approval no. 2022-109) and lasted three months. The two-hour interactive workshop was embedded into the reflection, integration, and assessment (RIA) week at the end of one of the organ system courses, which takes place in the winter semester of year 1 of the curriculum at Nova Southeastern University Dr. Kiran C. Patel College of Allopathic Medicine (NSU MD). The session was strategically placed in the curriculum after the students learned about sexually transmitted infections, practiced taking a patient’s medical sexual history, and were introduced to the use of MIs through problem-based learning cases. The skills and knowledge in these areas were beneficial for student preparedness for this workshop.

Participants

A total of 51 first-year medical students at the NSU MD participated in this workshop. Of them, 43 (84%) participated in the study by completing evaluations that included self-assessments.

Study/workshop design and facilitation

This interventional study employed pre- and post-evaluations to measure changes in participant perceptions of communication with MIs and Spanish-speaking LEP patients by engaging in an educational workshop. At the start of the workshop, facilitators discussed the learning objectives and participants were asked to complete a voluntary, anonymous pre-workshop evaluation (Tables 1-2). Learners then completed questions on their knowledge of factors that impact medical interpretation skills that were taken from *Spanish and the Medical Interview: A Textbook for Clinically Relevant Medical Spanish* [16] and adapted to fit our curriculum. The workshop featured four primary educational strategies: (1) facilitator-led instructional simulation activity, (2) interactive didactic component via PowerPoint presentation, (3) small-group activity, and (4) large-group interpretation simulation activity. The facilitator-led simulated activity exemplified best practices and limitations when eliciting a medical history with a Spanish-speaking LEP patient using an MI. At the conclusion of the encounter, facilitators guided the students to reflect on this activity. The interactive presentation started with introducing information on how language barriers can adversely affect quality of care and patient safety, and with the description of the Title VI of the Civil Rights Act of 1964, which affects persons with LEP [8]. Participants were then introduced to topics including when to use a professional MI, types of MIs, and best practices to work effectively with an MI. Furthermore, exercises were incorporated during this component: participants were asked to read a scripted dialogue between a physician and a nurse, exemplifying the use of a nurse as the ad hoc interpreter. The content of the evaluations was original and was developed by the authors to achieve the specific goals of the study.

**Table 1:** Pre-workshop participant evaluation: demographic information and language proficiency

Please indicate which ethnicity/race you identify with:
White
Black or African American
American Indian or Alaskan Native
Asian
Native Hawaiian or other Pacific Islander
Hispanic or Latinx
From multiple races
Other
I choose not to answer this question
Please indicate which gender you identify with:
Male
Female
Transgender
Non-binary/non-conforming
Other
I choose not to answer this question
Please select your age range (years):
24 or below
25–29
30–34
35–44
I choose not to answer this question
Rate your Spanish language fluency:
Fluent
Proficient
Limited
Little to none

**Table 2:** Pre-workshop participant evaluation: confidence assessment

Please rate how much confidence you have in your ability to...	No confidence			Complete confidence
	0	1	2	3
Explain the importance of using interpreters to improve limited English proficiency patient care.				
Explain the importance of laws requiring interpreters in healthcare.				
Recognize when a professional interpreter is needed for effective communication in clinical care.				
Employ medical Spanish vocabulary during a medical interview to elicit sexual history.				
Demonstrate respect for the individual and cultural humility when using interpreters.				

Additionally, a case scenario depicting the limitations and repercussions of using a family member as an interpreter was presented. Finally, participants engaged in a medical Spanish exercise where they practiced how to introduce themselves and the professional MI to the patient in Spanish during a patient encounter, followed by early stages of the medical interview.

Immediately following these activities, participants were divided into small groups. Participants were asked to read the case scenarios and associated questions, and then discuss their answers to the questions within their small groups. The case scenarios represented circumstances where on-site professional MI services were warranted to elicit the patient history and to provide the appropriate care to the patient. Participants were provided with a medical English-to-Spanish translation handout using translations from a clinically relevant medical Spanish textbook [16] as a primary source, with modifications to focus it on the case scenarios to aid them during this exercise.

In the segment of the workshop that followed, individual groups had the opportunity to simulate the case scenarios they previously discussed during the small-group activity. In short, participants simulated a physician, a Spanish-speaking LEP patient and a professional MI. After each simulation, all participants came together as a large group to discuss their reflection and reaction to the activity. The facilitator-guided discussion provided a transition to discuss key take-home points that emerged throughout the session. This activity highlighted how difficult it was to complete a task in a foreign language and the importance of utilizing a professional MI, and introduced best practices for utilizing a professional MI. As the workshop concluded, participants were asked to complete the anonymous post-workshop evaluation (Tables 3-5), which was used to evaluate the effectiveness of the workshop. The evaluation also included the self-assessment on their knowledge of factors that impact medical interpretation skills.

**Table 3:** Post-workshop participant feedback on objectives, content, and facilitation

Statement	Strongly disagree	Disagree	Neutral	Agree	Strongly agree
Workshop met objectives.					
Presentation was informative and included useful resources.					
Case discussions fostered my learning.					
The simulated cases were realistic.					
The facilitators were helpful in fostering discussions.					

**Table 4:** Post-workshop participant evaluation: confidence assessment

Please rate how much confidence do you have in your ability to…	No confidence			Complete confidence
	0	1	2	3
Explain the importance of using interpreters to improve limited English proficiency patient care.				
Explain the importance of laws requiring interpreters in healthcare.				
Recognize when a professional interpreter is needed for effective communication in clinical care.				
Employ medical Spanish vocabulary during a medical interview to elicit sexual history.				
Demonstrate respect for the individual and cultural humility when using interpreters.				

**Table 5:** Post-workshop participant reflection and feedback

Feedback
Please name two things that you will do/change as a result of this workshop.
What did you like best about the workshop?
What can we improve about the workshop?

Data analysis

An unpaired Student’s t-test was used to assess the statistical significance of the data obtained using GraphPad Prism, version 9.3.1 for Windows (GraphPad Software, San Diego, California, USA). A p-value less than 0.05 was considered as significant.

## Results

A total of 51 participants attended the workshop, of which 43 completed the pre- and post-workshop evaluations (Tables 1-5). The respondents self-identified their race/ethnicity as 20 (47%) White, 12 (28%) Asian, 4 (9%) Black or African American, 3 (7%) Hispanic or Latinx, 1 (2%) multiracial and Other 3 (7%). In terms of gender, 23 (53%) identified as male and 20 (47%) identified as female. Participants reported their self-evaluation on Spanish language fluency with 13 (30%) reporting little to no fluency, 23 (53%) limited fluency, 2 (5%) proficient fluency, and 5 (12%) being fluent in Spanish (Table 6).

**Table 6:** Demographic information and language proficiency of participants (n=43) who completed the pre- and post-workshop evaluation

		n (%)
Gender	Male	23 (53)
	Female	20 (47)
Age	≤24	30 (70)
	25-29	12 (28)
	30-34	1 (2)
Ethnicity	White	20 (47)
	Asian	12 (28)
	Black or African American	4 (9)
	Hispanic or Latinx	3 (7)
	Multiracial	1 (2)
	Other	3 (7)
Spanish Language fluency	Little to none	13 (30)
	Limited	23 (53)
	Proficient	2 (5)
	Fluent	5 (12)

Self-evaluation of participants regarding their confidence in their ability to meet the workshop’s learning objectives ranged from no confidence to complete confidence. Results from the unpaired Student’s t-test comparing the pre- and post-workshop results demonstrated that, for each objective, confidence improved, and the difference was statistically significant for three out of four learning objectives (Table 7).

**Table 7:** Mean responses to pre- and post-workshop evaluation confidence assessment (n=43) ^a^Rated on a four-point Likert scale with 0 indicating “no confidence” and 3 indicating “complete confidence”. A p-value from the unpaired Student’s t-test less than 0.05 was considered significant.

Objective	Mean pre-workshop evaluation^a^	Mean post-workshop evaluation^a^	p-value
Explain the importance of using interpreters to improve limited English proficiency patient care.	1.6	2.1	<0.05
Recognize when a professional interpreter is needed for effective communication in clinical care.	2.0	2.2	>0.10
Employ medical Spanish vocabulary during a medical interview to elicit sexual history.	0.6	1.5	<0.0001
Demonstrate respect for the individual and cultural humility when using interpreters.	2.0	2.4	<0.05

**Figure 1:**
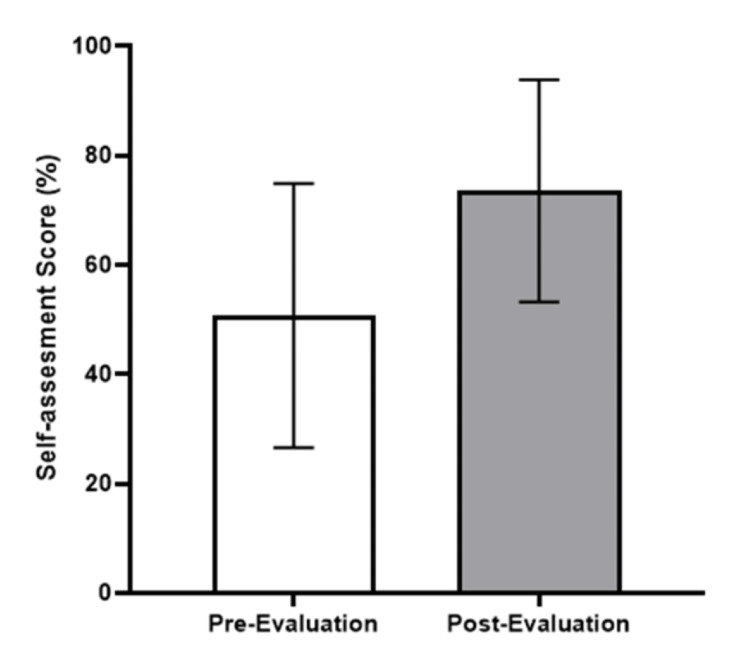
Self-assessment results from the pre- and post-workshop evaluation. An increase in the mean score was observed when comparing the pre-assessment mean score (51%) and the post-assessment mean score (74%)

In terms of the effect of the workshop on participants’ knowledge base, a comparison of pre- and post-workshop questionnaire responses on best practices when working with an MI showed an increase in the average score for the self-assessment (Figure 1). A statistically significant increase was seen in the question about the ideal position of the medical interpreter during a provider-patient interview (p<0.0001).

As shown in Table 8, a large majority of participants reported that the workshop met objectives, and the presentation was informative and included useful resources. In addition, they also agreed that the case discussions fostered their learning, the simulated cases were realistic, and the facilitators were helpful in fostering discussions.

**Table 8:** Summary of participant responses to post-workshop feedback on objectives, content, and facilitation (n=43)

Statement	Number of responses (%)		
	Disagree or strongly disagree	Neutral	Agree or strongly agree
Workshop met objectives.	0 (0)	1 (2)	42 (98)
Presentation was informative and included useful resources.	0 (0)	2 (5)	41 (95)
Case discussions fostered my learning.	1 (2)	3 (7)	39 (91)
The simulated cases were realistic.	0 (0)	2 (5)	41 (95)
The facilitators were helpful in fostering discussions.	0 (0)	4 (9)	39 (91)

Participants were asked to provide written responses to indicate changes they planned to implement because of the workshop, and a number of compelling themes emerged from these comments (Table 9). After attending the workshop, many participants shared their awareness on their language limitations and when an MI is needed. They also shared ways they would implement what they learned to better utilize MIs in the clinical setting with LEP patients. In addition, several participants expressed their intent to use content and resources from the workshop as the starting point for improving their medical Spanish vocabulary.

**Table 9:** Summary of participant responses to “Please name two things that you will do/change as a result of this workshop.”

Theme	Representative quotes
Medical interpreter need	“I now understand the role of a medical interpreter and when they should be used.”
	“More readily call upon the assistance of interpreters.”
	“Be more aware of when an interpreter might be needed.”
	“Offer a patient a medical interpreter even if they have a family member available.”
	“Using a medical interpreter when I encounter language barriers between myself and patients.”
Medical interpreter practice	“Use short sentences when using an interpreter.”
	“Understand how to position an interpreter in a conversation with a patient.”
	“Make sure to talk directly to the patient and make eye contact.”
	“Address the patient directly when an interpreter is used.”
Medical Spanish knowledge	“Study common phrases (in Spanish) to explain certain basic situations to a patient during those moments we are waiting for an interpreter.”
	“Practice my medical Spanish vocabulary more.”
	“Understand the medical terminology in Spanish more thoroughly.”

Participants also provided valuable insights into how to improve the workshop. Participants suggested future workshops should also include videos portraying a clinical encounter of a physician using a professional MI to provide care for an LEP patient. Several participants would have liked more resources to learn medical Spanish, with responses such as “Perhaps have more videos on professional interpreters and discuss their techniques” and “Having more resources that people can use to learn more about medical Spanish.”

The most common remark regarding what was most appreciated about the workshop was its interactivity (between the students and with the facilitators), simulation activities, case studies, and the opportunity to learn medical Spanish phrases, with responses such as “The examples of the students in the class acting out and then discussing their performance,” “I liked how engaging it was and how I was able to practice my Spanish,” and “I enjoyed the cases and the role playing that was involved.”

## Discussion

As the population of individuals and families with LEP continues to expand in the United States, physicians will need to be better prepared to care for growing numbers of patients with LEP and to understand the issues pertaining to communication with this population. The existing literature has documented the deficiencies in physicians’ effectiveness in medical encounters with LEP patients, indicating the importance of improving medical training in this domain [17]. Additionally, a study that surveyed medical students found that only 20% of them reported being very well or well prepared to provide care to LEP patients [18]. Therefore, we developed and implemented a workshop as an early intervention to teach first-year medical students the skills and best practices on how to communicate effectively with MIs and Spanish-speaking LEP patients. This workshop fills an important gap in UME and provides a comprehensive orientation to interpretation resources. It is important to begin the training of medical students on this topic early in their training with the potential to reinforce this information at a later time in the curriculum. Many of the institutions that offer a formal curriculum to train students to work with MIs and/or patients with LEP do so during the first two years of medical school, with a few schools spanning their training across two to four years [10].

The workshop was well received by the participants who reported that the workshop met its objectives; the presentation was informative and included useful resources; the case discussion fostered their learning and the simulated cases were realistic. A comparison of pre- and post-evaluation data showed that the workshop facilitated statistically significant improvements in the confidence of participants to explain the importance of using interpreters to improve limited English proficiency patient care, to employ medical Spanish vocabulary during a medical interview to elicit sexual history, and to demonstrate respect for the individual and cultural humility when using interpreters. Although the self-assessment test on their knowledge of factors that impact medical interpretation skills showed an increase in the average score, the increased scores for some questions were not statistically significant. This implication may indicate that the participants already had prior knowledge, for instance, from personal experiences and/or from other parts of the curriculum such as case-based learning curriculum that addresses this content. The post-workshop item responses also provided important continuous quality improvement data for areas that can be addressed further in future iterations of the workshop and/or later in the curriculum as part of the longitudinal delivery of these topics.

As demonstrated by the qualitative feedback received, participants had increased awareness of their language limitations and of when a professional MI is needed to care for LEP patients. The feedback also highlighted best practices on communication skills participants can use when interacting with an LEP patient using a professional MI. Moreover, participants emphasized the importance of understanding and learning more medical Spanish. As the predominant and fastest growing non-English language in the United States, many medical schools have reported having a medical Spanish curriculum. However, the curricula are not consistently linked to learner assessment [19]. An assessment should be required to demonstrate Spanish language proficiency and should incorporate communication safeguards to prevent errors.

There were also some limitations present in our study. First, our sample size was small due to class size. However, the demographics and Spanish proficiency of the participants were comparable to other programs in the United States. Second, due to time constraints, 6 out of 10 small groups had the opportunity to simulate the case scenarios provided. We ensured that there were substantial reflection, feedback and discussion opportunities for the groups that participated. Finally, we utilized Spanish-speaking faculty and staff with experience in this area, including a practicing clinician but we did not make use of professional MIs during the simulation activities. We will aim to include professional MIs in future iterations of the workshop as they can also provide their direct experiences and insights during teaching and discussion.

## Conclusions

The results from our study suggest that the implementation of MI training in the undergraduate medical curriculum can aid in the development of medical students equipping them with the skills and best practices to communicate effectively with professional MIs and Spanish-speaking LEP patients. This workshop focused on Spanish-speaking LEP patients, but this design can be adapted for any LEP patient population. Though not directly measured, we expect this intervention to have improved quality of care for LEP patients through professional interpreter services.
